# Racial Disparities in Hypertension-Related Hospital Mortality Among Adults in the United States

**DOI:** 10.7759/cureus.81043

**Published:** 2025-03-23

**Authors:** Gideon U Noah, Mercy U Omohoro, Hezborn M Magacha, Catherine D Fuko, Tobechukwu Ezike

**Affiliations:** 1 Internal Medicine, Center of Excellence in Inflammation, Infectious Disease, and Immunity, East Tennessee State University, Johnson City, USA; 2 Health Sciences, Kent State University Ohio, Kent, USA; 3 Internal Medicine, East Tennessee State University Quillen College of Medicine, Johnson City, USA; 4 Public Health, East Tennessee State University, Johnson, USA; 5 Epidemiology and Public Health, College of Public Health, East Tennessee State University, Johnson City, USA

**Keywords:** cardiology, cardiovascular mortality, hypertension, public health problems, racial disparity

## Abstract

Background and objectives

Hypertension has been known to be a significant contributor to hospital admissions and, consequently, high mortality rates across the globe and in the United States. Racial disparities, which entail the unequal treatment or outcomes experienced by different racial or ethnic groups, influenced by systemic inequality and/or discrimination, have been shown by other studies to have an impact on hypertension-related in-hospital mortality. These disparities may have influenced several factors that impact equal rights and access to healthcare across racial groups. For example, the poor implementation of health policies, poor access to care, and poor socioeconomic factors that highlight the presence of racial disparities may have a huge impact on hypertensive care. This research paper builds on existing knowledge and aims to further explore the legitimacy of the claim that racial disparities exist among different racial groups, particularly among adults in the United States, and that it plays a major role in causing in-hospital mortality. The data used for this study were drawn from the Healthcare Cost and Utilization Project’s (HCUP) National Inpatient Sample (NIS) between 2016 and 2022.

Methods

Using the NIS database, we identified 360,642 individuals who were aged 18 years and older and were hospitalized for hypertension-related issues in a hospital within the United States. Descriptive, bivariate, and multivariate analyses were conducted to examine the relationship between race and in-hospital mortality. Potential confounders were adjusted for and included age, sex, location of hospital, insurance payer, and diabetes (as a comorbidity).

Results

In our study sample, 241,991 (67%) of the patients were White, 72,778 (20%) were Black, 29,464 (8%) were Hispanic, 6,564 (2%) were Asian/Pacific Islanders, 1,623 (1%) were Native Americans, and 8,187 (2%) were individuals from other races that were not included in the races mentioned above. For this study population, significant racial disparities in hypertensive in-hospital mortality were observed, with Black patients experiencing higher mortality rates (55%) compared to their White counterparts (OR=1.55, 95% CI=1.42,1.69, p<0.0001).

Conclusion

The findings of this study provide further evidence of racial disparities in hypertension-related hospital mortality, with Black individuals showing significantly higher odds of hospital mortality from hypertension. This study highlights the need to combat health inequities in hypertension outcomes through a targeted approach, which could include enhancing access to high-quality hypertensive care, provision of culturally sensitive care, and promotion of positive policies that can enhance these outcomes.

## Introduction

Hypertension is a chronic medical condition associated with an increased risk of cardiovascular complications, including heart failure and stroke, as well as other severe health outcomes that may contribute to premature mortality [1,2]. Despite targeted public health efforts, the rates of hypertension remain persistently high, and the prevalence of hypertension control is low in the United States [2]. Hypertension-related hospital mortality rates are significantly affected by a range of factors that may include demographic, socioeconomic, or healthcare-related factors such as age, race, sex, presence of diabetes, insurance payer, and location of the hospital of care. A study conducted by the National Center for Health Statistics (NCHS) found that hypertension-related mortality was higher in women than men for those aged 85 and older, while it was lower in women aged 45-84 during the 2000-2013 period [3,4]. While the age in focus for the study by the NCHS captures patients above 45 years, it gives us insight on what to expect from a segment of our study population and invariably leaves a gap that our study intends to fill. This study by the NCHS was important in identifying the fact that the sex and age of patients could have an impact on hypertensive mortality rates. Other studies identified overall death rates in rural areas to be higher than those in urban areas when analyzed based on the top 10 causes of death in the United States [4]. This could be attributed to poor living conditions or poor access to high-quality care. Living in a resource-limited area and only getting access to rural healthcare facilities have been associated with a higher prevalence of hypertension when compared to living in higher socioeconomic neighborhoods. Living in these resource-limited areas is often accompanied by a reduction in hypertension control and consequently, higher rates of secondary heart disease, renal failure, stroke, and death [5].

Racial disparities have been identified to play an important role in causing a rise in the rate of hypertension-related hospital mortality in U.S. adults. Its impact on limiting healthcare access across racial groups, its impact on influencing healthcare provider biases, and its negative influence on social determinants of health have been identified [6]. In a study conducted to determine how racial disparities affect hypertension-related hospital mortality, it was observed that following stratification of age-adjusted mortality rates (AAMRs), death rates were highest among non-Hispanic Black or African American patients, followed by Non-Hispanic American Indian or Alaska Native, Hispanic, or Latino, non-Hispanic Asian or Pacific Islander, and Non-Hispanic White populations. The overall AAMR shows that 45.7 deaths occur in 100,000 non-Hispanic Black or African Americans, 24.7 deaths in non-Hispanic American Indians or Alaska Natives, 23.4 deaths in Hispanic or Latinos, 19.3 deaths in non-Hispanic Asians or Pacific Islanders, and 15.4 deaths in non-Hispanics in 100,000 patients [7]. A hospital-based cross-sectional study was conducted between July 2020 and February 2021 in 22 tertiary medical college hospitals in Bangladesh including adults >18 years. The study showed that diabetes was found to be the most prevalent (47.6%) comorbid condition among hypertensive patients, while obesity was the second (32.3%), followed by heart disease (16.2%), visual impairment (15%), and neurological disease (13.8%) [8]. Based on the same study by Rahman et al., males were found to have more comorbid conditions, showing higher percentages in all types of comorbidities, compared with females [8]. The Bangladesh study was included in this research despite its geographical and demographic differences from the United States because it provides valuable insights into comorbidities that influence hypertension outcomes. While the population in Bangladesh differs from that of the United States in terms of racial composition, healthcare access, and socioeconomic conditions, the study's findings on diabetes and other coexisting conditions as significant risk factors for hypertension-related mortality are biologically relevant across diverse populations. Despite the difference in location of this study from ours, its findings should be interpreted within the context of the U.S. healthcare system and population-specific disparities.

A study conducted by Raja et al. highlighted the rate of hypertension-related hospital mortality to be higher in men than in women after AAMR stratification among different U.S. adult races [9]. Although these studies have highlighted some factors that might cause these disparities in hypertension-related hospital deaths, we must investigate whether other factors such as insurance payer, comorbidities, and location and type of hospital influence these rates. It is perceived that insurance status could have a significant influence on healthcare access, quality, and outcomes. This is because the type of insurance a patient has might often determine the quality and type of preventive and specialist care that they can access. The location and type of hospital also weighs in on the level of specialist care that patients can access. An understanding of the factors that contribute to why there is a higher mortality rate in a specific racial group than others will help provide a foundation for formulating evidence-based interventions and control measures aimed at achieving a reduction in racial disparities associated with hypertension-related hospital mortality rate among U.S. adults.

This study aims to identify the presence of notable differences in in-hospital mortality rates between racial and ethnic groups and to explore potential factors that contribute to these disparities. The goal of this study is to provide insights for public health initiatives, policies, and interventions that are aimed at reducing racial disparities in hypertensive care and improving health outcomes for all adults with hypertension.

## Materials and methods

This study was conducted in the United States, a nation notable for its vast geographical diversity and considerable demographic heterogeneity. In 2023, the United States had an estimated population of approximately 334.9 million.

The study utilized data from the Healthcare Cost and Utilization Program’s (HCUP) National Inpatient Sample (NIS) for 2016-2022. The NIS database is a nationally representative sample of patient discharges from acute care hospitals in the United States. It is the largest all-payer inpatient database in the United States and provides weighted estimates for national trends. To ensure that the study sample was representative of the target population, the sample was weighted using a weighting variable. The sample size for this analysis is 360,641 and included patients aged 18 years and above who were hospitalized for hypertension. Patients who were less than 18 years and had no diagnosis of hypertension were excluded.

The variables used include mortality, race, age, sex, presence of diabetes, location and type of hospital, insurance payer, and a weighting variable. The mortality variable (served as hospitalized hypertensive mortality for this study) served as the primary outcome variable and categorized as either “died” or “did not die.” The variable “race” was the primary predictor variable and was categorized as 1=White individuals, 2=Black individuals, 3=Hispanic individuals, 4=Asian or Pacific Islander individuals, 5=Native American individuals, and 6=Others (which includes multiracial patients and those who do not fall into any of the other racial categories). The age variable was re-coded into five categories: 1=18-34, 2=35-49, 3=50-64, 4=65-74, and 5=75+ years. Gender was categorized as 0=male and 1=female. The diabetes mellitus “DM” variable (uncomplicated diabetes mellitus) was recoded into two categories: 1=comorbidity is not present and 2=comorbidity is present. The variable “hospital location" was recoded into three categories: 1=rural, 2=urban non-teaching, and 3=urban teaching (Table 1). The insurance variable was categorized into the following: 1=Medicare, 2=Medicaid, 3=private insurance, 4=self-pay, 5=no charge, and 6=others (which captured military/VA, state programs, workers' compensation, and other forms of insurance that were not mentioned in the above categories). Based on previous studies that were reviewed, the above variables were used as the potential confounders for this study.

**Table 1:** Summary of variables, original response options, and recoded response options

Variable Name	Variable Description	Original Response Options	Recoding Plan	Recoded Response Options
AGE	Age of years of participants at the time of admission	0-124 = Age in years	(.), (A), (B), (C) were recoded as missing with age(years) split into six categories	1=18-34
		(.)=Missing		2=35-49
		A=Invalid		3=50-64
		B=Unavailable from source		4=65-74
		C=Inconsistent		5=75+
DIED	Patients who died during hospitalization	0=Did not die	(.), (A), (B) were recoded as missing	0=did not die
		1=Died		
		(.)=Missing		
		A=Invalid		
		B= Unavailable from source		1 = died
FEMALE	Indicator of sex	0=Male	(.), (A), (C) were recoded as missing	0=Male
		1=Female		
		(.)=Missing		
		A=Invalid		1=Female
		C=Inconsistent		
RACE	Race/ethnicity of patient	1=White	(.), (A), (B) were recoded as missing	1=White
		2=Black		2=Black
		3=Hispanic		3=Hispanic
		4=Asian/Pacific Islanders		4=Asian/Pacific Islanders
		5=Native American		5=Native American
		6=Other		6=Other
		(.)=Missing		
		A=Invalid		
		B=Unavailable from source		
HTN	Comorbidity measure that codes hypertension (combine uncomplicated and complicated)	0=Comorbidity is not present	(A) was recoded as missing	0=Comorbidity is not present
		1=Comorbidity is present		
				1=Comorbidity is present
		A=Invalid		
DM	Comorbidity measure for diabetes, uncomplicated	0=Comorbidity is not present	(A) was recoded as missing	0=Comorbidity is not present
		1=Comorbidity is present		
				1=Comorbidity is present
		A=Invalid		
PAY1	Insurance payer	1=Medicare	(.), (A), (B) were recoded as missing	1=Medicare
		2=Medicaid		2=Medicaid
		3=Private Insurance		
				3=Private Insurance
		4=Self-pay		
		5=No charge		
		6=Other		4=Self-pay
		(.)=Missing		
				5=No charge
		(A)=Invalid		
				6=Other
		(B)=Unavailable		
HOSP_LOCTEACH	Location and teaching status of hospital	1=Rural	Missing values (.) were erased	1=Rural
		2=Urban non-teaching		
				2=Urban non-teaching
		3=Urban teaching		
				3=Urban teaching
		(.)=Missing		

Analysis was conducted using SAS statistical software version 9.4 (SAS Inc., Cary, NC). Descriptive analysis was conducted for all the variables. The chi-square test was used to test the significance of the differences in proportion between the groups. Bivariate analysis, specifically, simple logistic regression, was conducted to assess the relationship between each of the predictor variables and the outcome variable. A multivariate analysis was carried out with the multivariate logistic regression model and adjusted for clustering to assess the association between the primary predictor, other predictors, and the outcome variable. Sensitivity testing was conducted, and outliers were excluded. Controlling these variables offered an insight on the relationship between race/ethnicity and hypertension-related in-hospital mortality.

## Results

Descriptive analysis

Most of the participants were 75 years and above, accounting for 161,279 (44.72%) of the study population, with 87,600 (24%), 79,522 (22%), 28,346 (8%), and 3,895 (1%) making up the population of patients aged 50-64 years, 65-74 years, 35-49 years, and 18-34 years, respectively. Out of the study population, 6,167 (1.7%) of them died of hypertension (Table 2). There were more females in the study, making up 184,829 (51%) of the study population (Table 2). Diabetes was found to be a comorbidity in only 149,378 (41%) of the study population. Most of the study participants attended urban teaching hospitals in the country signified by the 242,712 (67.3%) of the population that were identified to have attended such hospitals. Most of the participants used Medicare as their insurance payer (248,699; 68.96%), with an almost equal number of participants paying with either private insurance or Medicaid. In the chi-square analysis, all relationships were significant at p<0.05.

**Table 2:** Descriptive analysis of hypertension-related hospital mortality from the NIS dataset (2016–2020) (N = 360,642)

Variable	Characteristics	n (%)
Age (years)	18-34	3,895 (1.08)
	35-49	28,346 (7.86)
	50-64	87,600 (24.29)
	65-74	79,522 (22.05)
	75+	161,279 (44.72)
Died (due to hypertension)	No (alive)	354,475 (98.29)
	Yes (dead)	6,167 (1.71)
Race	White	241,991 (67.10)
	Black	72,778 (20.18)
	Hispanic	29,464 (8.17)
	Asian/Pacific Islander	6,564 (1.82)
	Native American	1,623 (0.45)
	Other	8,187 (2.27)
Sex	Male	175,813 (48.75)
	Female	184,829 (51.25)
Diabetes	Present	149,378 (41.42)
	Absent	211,264 (58.58)
Location of teaching hospital	Rural	37,038 (10.27)
	Urban non-teaching	80,928 (22.44)
	Urban teaching	242,712 (67.30)
Insurance payer	Medicare	248,699 (68.96)
	Medicaid	44,323 (12.29)
	Private insurance	45,044 (12.49)
	Self-pay	14,354 (3.98)
	No charge	1,010 (0.28)
	Other	7,177 (1.99)

Upon using simple logistic regression to assess the relationship between predictor variables and the outcome variable, it was seen that White patients died the most during hospitalization, followed by Blacks and Hispanics (Table 3). According to age, the highest mortality rate was seen in the age group of 75+ years, with 1.2% of that population dying during hospitalization (Table 3). More females died than males, with 0.9% and 0.8% mortality rates, respectively. Participants who also had diabetes were assessed, and there were less deaths in those who had diabetes as a comorbidity. The location of the hospital attended by the participants also seemed to have had an influence on the mortality rates as there were more deaths among those who attended urban teaching hospitals (1.2%). In terms of insurance payment, those who pay their insurance with Medicare were seen to have the highest mortality rates, with 1.3% of that population dying (Table 3).

**Table 3:** Frequency distribution of predictor variables by in-hospital mortality The chi-square test was used to determine the p-values

Variable	Value	Died (%)		X^2^	p-Value
		Yes	No		
Race	White	1.3	65.8	1.0	<0.0001
	Black	0.2	20.0		
	Hispanic	0.1	8.1		
	Asian/Pacific Islander	0.02	1.8		
	Native American	0.008	0.4		
	Other	0.04	2.2		
Age	18-34	0.008	1.1	1.0	<0.0001
	35-49	0.05	7.8		
	50-64	0.2	24.1		
	65-74	0.3	21.8		
	75+	1.2	43.5		
Sex	Female	0.9	50.3	1.0	<0.0001
	Male	0.8	47.9		
Diabetes	Present	0.6	40.8	1.0	<0.0001
	Absent	1.1	57.4		
Hospital location	Rural	0.2	10.1	1.0	<0.0085
	Urban non-teaching	0.4	22.1		
	Urban teaching	1.2	66.1		
Insurance payer	Medicare	1.3	67.6		
	Medicaid	0.1	12.2		
	Private Insurance	0.2	12.3	1.0	<0.0001
	Self-pay	0.03	3.9		
	No charge	0.001	1.9		
	Others	0.06	1.9		

Bivariate analysis was conducted, which revealed that Black patients (OR=2.28, 95% CI=2.10-2.47), adults in the 35-49 age group (OR=1.20, 95% CI=0.8-1.8), males (OR=1.11, 95% CI=1.05-1.16), insurance payers with no charge (OR=1.62, 95% CI=0.67-3.69), and urban non-teaching hospitals (OR=1.15, 95% CI=1.05-1.27) had higher odds of in-hospital mortality when compared to their counterparts (Table 3). Participants who did not have diabetes as a comorbidity had a 29% lower likelihood of dying during hospitalization (OR=0.71, 95% CI=0.67-0.75) (Table 4).

**Table 4:** Bivariate and multivariate analysis for odds ratio, confidence intervals, and p-values for hypertensive in-hospital mortality

Variable	Values	Unadjusted Odds Ratio (95% CI)	p-Value	Adjusted Odds Ratio (95% CI)	p-Value
Race	White	Ref		Ref	
	Black	2.28 (2.10–2.47)	<0.0001	1.55 (1.42–1.69)	<0.0001
	Hispanic	1.42 (1.29–1.58)	<0.001	1.18 (1.06–1.31)	<0.01
	Asian/Pacific Islander	1.22 (1.01–1.48)	<0.05	1.15 (0.95–1.40)	0.158
	Native American	1.21 (0.83–1.78)	0.3274	0.92 (0.63–1.35)	0.668
	Other	1.22 (1.03–1.45)	<0.05	1.08 (0.91–1.29)	0.387
Age	18-34	Ref		Ref	
	35-49	1.20 (0.8–1.8)	0.3781	1.24 (0.83–1.86)	0.296
	50-64	0.88 (0.60–1.28)	0.5084	0.91 (0.63–1.34)	0.624
	65-74	0.54 (0.37–0.79)	<0.01	0.53 (0.36–0.78)	<0.01
	75+	0.27 (0.19–0.39)	<0.001	0.27 (0.18–0.40)	<0.001
Sex	Female	Ref		Ref	
	Male	1.11 (1.05–1.16)	<0.001	0.94 (0.89–0.99)	<0.05
Diabetes	Present	Ref		Ref	
	Absent	0.71 (0.67–0.75)	<0.0001	0.81 (0.77–0.86)	<0.001
Insurance payer	Self-pay	Ref		Ref	
	Medicare	0.4 (0.34–0.50)	<0.0001	1.10 (0.90–1.34)	0.348
	Medicaid	0.99 (0.81–1.23)	0.925	0.95 (0.77–1.17)	0.631
	Private Insurance	0.55 (0.45–0.68)	<0.001	0.79 (0.65–0.97)	<0.05
	No charge	1.62 (0.67–3.69)	0.268	1.65 (0.67–4.05)	0.2752
	Other	0.25 (0.20–0.31)	<0.0001	0.97 (0.89–1.05)	<0.05
Hospital location	Rural	Ref		Ref	
	Urban non-teaching	1.15 (1.05–1.27)	<0.05	1.13 (1.02–1.24)	<0.05
	Urban teaching	1.07 (0.99–1.16)	0.0942	0.97 (0.89–1.05)	0.47

Although from the simple logistic regression, the in-hospital mortality rates were seen to be highest in White patients, after accounting for all the other variables and adjusting the odd ratios, Blacks were seen to have a 55% higher likelihood of mortality during hospitalization than their White counterparts (OR=1.55, 95% CI=1.42-1.69). As a matter of fact, upon this adjustment, all racial groups, with the exception of Native Americans, exhibited a significantly higher likelihood of mortality, while the association for Native Americans was not statistically significant. Participants in the age group of 75+ had the highest percentage of deaths and the lowest adjusted odds ratio (OR=0.27; 95% CI=0.18-0.40). This points to the fact that while they make up the population with the most deaths, when adjusted for other variables, they show lesser relative risk compared to the other age groups. Participants with diabetes as a comorbidity were 19% less likely to die during hospitalization (OR= 0.81, 95% CI=0.77-0.86). While hypertensive males were perceived to have a higher mortality rate, when the odds ratio was unadjusted, being male showed a slightly protective effect. Based on insurance payer, self-pay was used as the reference, and upon adjustment of the odds ratio, Medicare patients had increased risk of mortality (OR=1.10, 95% CI=0.90-1.34). However, Medicaid and private insurance patients show no significant variation. The "No charge" group has a wide confidence interval, indicating variability. Participants who attended urban non-teaching hospitals (OR=1.13, 95% CI=1.02-1.24) showed 13% more likelihood of in-hospital mortality when compared to those who presented to rural hospitals who had the lowest mortality rates.

## Discussion

In this study, we examined hypertensive patients who have been hospitalized. We wanted to determine the racial disparities in in-hospital mortality, if any, in this category of patients and explore other differences reported by age, sex, hospital location, presence of obesity as a comorbidity, and insurance payer.

Our results showed that while White patients make up the higher population of hypertension-related in-hospital deaths, Blacks and Hispanics have higher adjusted mortality odds. This is consistent with previous studies that have identified racial disparities in hypertension prevalence, treatment, and outcomes [2,7,10]. Howard et al. determined the prevalence of hypertension and its control in the United States. They found that non-Hispanic Blacks had higher prevalence of hypertension and that they were less likely to have it controlled when compared to their White counterparts. This disparity in hypertension control could possibly be a contributing factor to the disparity seen in hospital mortality rates because poorly controlled hypertension has the potential to lead to various negative health outcomes including mortality. Also, disparities in healthcare access and quality of care may play a role. Studies have shown that non-Hispanic blacks have more likelihood of facing barriers to accessing healthcare or even quality care including lower insurance coverage and less access to primary care providers [11].

Increased risk of hypertensive in-hospital mortality was observed in Blacks and Hispanics and can be due to several factors that might function in isolation or in connection with each other. These factors may include delayed diagnosis, inadequate access to healthcare, lower adherence to treatment, and the impact of structural racism on health outcomes [12]. The Joint National Committee report of 1997 [1] highlighted and buttressed the need for early detection and treatment of hypertension; however, the persistent presence of disparities in treatment outcomes shows that the measures that were put in place have not been equally effective across all racial groups.

The findings of this study suggest that hypertension-related hospital mortality increases with age, with individuals in the age group of 75+ years having the highest mortality. However, when the odds ratio was adjusted and other factors put into consideration, this age group had lower relative mortality rates. This drop may possibly be due to survival bias (i.e., frail individuals dying before hospitalization) or differential hospitalization patterns. This suggests that age alone does not account for these disparities. This is in line with research by Shah et al., which found that cardiometabolic mortality trends shifted with age, but racial disparities remained constant across all age groups [6].

Pertaining to gender, our study initially suggests that men have higher mortality rates, but this observation changes when the odds are adjusted. Previous studies have shown that men have a higher prevalence of hypertension and poorer control of it when compared to women [6]. This may be a huge contributor to this trend seen in our study. However, Ostchega et al. noted that this relationship is moderated by biological and behavioral factors like hormonal influences and difference in healthcare-seeking behavior [2].

The findings in our study observed the higher hypertensive in-hospital mortality rates in diabetics, which aligns with studies that show that diabetes worsens hypertension-related cardiovascular risks [7]. This interconnectedness between diabetes, hypertension, and cardiovascular mortality as documented in these studies places an emphasis on the need to employ a comprehensive chronic disease management strategy in combatting this. More aggressive approach towards comprehensive, holistic patient management strategies and better surveillance needs to be implemented.

Our findings show that rural hospitals have lower mortality rates when compared with urban non-teaching hospitals (OR=1.13, 95% CI=1.02-1.24). This finding is worthy of note seeing that urban hospitals serve a more diverse and medically complex population than rural hospitals [12]. They also provide greater access to specialists, advanced treatment protocols, and better ICU care [12]. Although urban teaching hospitals are perceived to see more variety of complex cases than rural and urban-non-teaching hospitals, they do not show a significantly increased mortality rate. This may be accrued to better availability of resources and more specialized care. Furthermore, after adjustment (OR=0.97, p = 0.47), the effect of urban teaching hospitals was non-significant, indicating that the initially observed higher mortality rate may be attributed to other factors such as case severity rather than hospital type alone.

The insurance status of the patients showed a significant impact on mortality rates. With self-pay patients serving as the reference group, Medicare patients exhibited a higher adjusted mortality risk (OR=1.10, 95% CI=0.90-1.34). The confidence interval including 1.34 signifies its non-significance. This finding is in agreement with previous studies that suggest that lower-income and uninsured populations face higher barriers to hypertension management [6]. The “no charge” group exhibits highly variable mortality risk due to a small sample size; however, this observation aligns with previous studies that suggested that uninsured patients receive fragmented emergency care [10].

Addressing in-hospital mortality among hypertensive adults with particular focus on balancing racial disparities would require a robust approach. Improving policies that can improve access to healthcare across all racial groups can be a solution. Implementing policies such as expanding Medicaid eligibility, increasing the funding for community health centers, and increasing bonuses and incentives for healthcare providers to work in underserved areas can help improve not only healthcare access but also the number and quality of healthcare providers in underserved areas. Also, healthcare providers can be made to undergo cultural competency training (for example, implicit bias training and recruitment of a racially diverse population of healthcare professionals) to stay abreast of the cultural intricacies of the population they are catering to. This will help them better understand the unique needs of the population and offer the best care possible [10]. Hypertension management can be enhanced by implementing evidence-based guidelines for the management of hypertension. These guidelines should be universal to all ethnic and racial groups and delivered without bias or prejudice. This may include blood pressure monitoring, medication management, and lifestyle interventions such as dietary counselling and lifestyle modification (e.g., diet and exercise) [12,13]. Patient education and empowerment will enable patients to take up active roles in the management of their hypertension. This can include provision of culturally appropriate educational materials, offering support for medication adherence, and providing patient-centered care models that place emphasis on patient’s values and ethnic backgrounds [14]. Implementation of these evidence-based guidelines may be met by certain barriers such as medication affordability and physician adherence; however, potential solutions such as insurance coverage for antihypertensive medications and policies to ensure physician compliance can help abate this [15]. To address the issue of hypertension, which is a public health issue of growing concern, it is important to consider both healthcare policy changes and individual targeted interventions that can help to reduce disparities and improve outcomes for all patients.

Strengths and limitations

The statistical analysis approach used for this study, such as the use of descriptive, bivariate, and multivariate regression analysis, provides a robust strategy that effectively captures the relationship between race and in-hospital mortality among adults with hypertension. The multivariate analysis allowed for control (through adjusted models) of potential confounders such as age, sex, and comorbidity, which could possibly have influenced the relationship between race and in-hospital mortality. Investigating the impact of racial disparities on health outcomes is an important and relevant topic in healthcare research, and, thankfully, this study adds to the understanding of racial disparities in hypertensive care.

One of the limitations of this study is that we relied on self-reported data for race, which may not be accurate or complete. Secondly, the data reported was of only hospitalized individuals in the United States, and thus the results may not be applicable to the general population. This is so because disease severity might differ between those hospitalized and those managed in outpatient settings. Lastly, our study focused on hospital mortality but did not account for deaths outside the hospital after discharge. A recommendation for this would be to carry out longitudinal follow-up studies that would provide a more comprehensive understanding of mortality trends. Potential biases could also arise from misclassification of the different variables or inconsistency in racial identity reporting.

## Conclusions

In this study, adjusted analyses showed significantly higher odds of in-hospital mortality for Black and Hispanic patients, while other racial groups showed varied results. This highlights the differences in healthcare outcomes based on race and suggests the need for efforts to be streamlined in line with racially focused interventions. These interventions will go a long way in the fight to abate or at least minimize hypertensive mortality among adults, making sure that they receive high quality care regardless of their ethnic origin.

The findings of this study are important in fostering the creation and implementation of specific interventions that target adult hypertensives particularly, non-Hispanic Blacks. This could involve improving access to high-quality hypertensive care through enhanced hospital care protocols, creation of culturally responsive care, and the implementation of policies that can help to achieve this. The stakeholders, healthcare providers, policymakers, and advocacy organizations can use this information to create focused interventions. Healthcare providers can implement culturally sensitive hypertensive care educational programs and follow universal hypertensive management guidelines. Policy-makers can improve on policies that widen Medicaid coverage (which has shown positive impact on hypertension outcomes), increase funding to community-based hospitals, and increase the incentives offered to healthcare providers (e.g., loan forgiveness, salary adjustments, and better recruitment programs) who are willing to work in undeserving areas. Advocacy groups can raise more awareness about racial disparities in hypertensive care and advocate for policies that address these disparities. Policies such as public awareness campaigns or legal actions to enforce healthcare equity will be impactful. An integrated effort by stakeholders can foster enhancement of hypertensive care and ultimately reduce the racial disparities associated with hypertensive outcomes.
